# RNA-Binding Proteins Impacting on Internal Initiation of Translation

**DOI:** 10.3390/ijms141121705

**Published:** 2013-11-01

**Authors:** Encarnación Martínez-Salas, Gloria Lozano, Javier Fernandez-Chamorro, Rosario Francisco-Velilla, Alfonso Galan, Rosa Diaz

**Affiliations:** Centro de Biología Molecular Severo Ochoa, Consejo Superior de Investigaciones Científicas-Universidad Autónoma de Madrid, Cantoblanco, Madrid 28049, Spain; E-Mails: glozano@cbm.uam.es (G.L.); jfernandez@cbm.uam.es (J.F-C.); rfrancisco@cbm.uam.es (R.F-V.); agalan@cbm.uam.es (A.G.); rdiaz@cbm.uam.es (R.D.)

**Keywords:** RNA-binding proteins, internal initiation of translation, IRES elements

## Abstract

RNA-binding proteins (RBPs) are pivotal regulators of all the steps of gene expression. RBPs govern gene regulation at the post-transcriptional level by virtue of their capacity to assemble ribonucleoprotein complexes on certain RNA structural elements, both in normal cells and in response to various environmental stresses. A rapid cellular response to stress conditions is triggered at the step of translation initiation. Two basic mechanisms govern translation initiation in eukaryotic mRNAs, the cap-dependent initiation mechanism that operates in most mRNAs, and the internal ribosome entry site (IRES)-dependent mechanism activated under conditions that compromise the general translation pathway. IRES elements are *cis*-acting RNA sequences that recruit the translation machinery using a cap-independent mechanism often assisted by a subset of translation initiation factors and various RBPs. IRES-dependent initiation appears to use different strategies to recruit the translation machinery depending on the RNA organization of the region and the network of RBPs interacting with the element. In this review we discuss recent advances in understanding the implications of RBPs on IRES-dependent translation initiation.

## 1. Introduction

**Figure 1:**
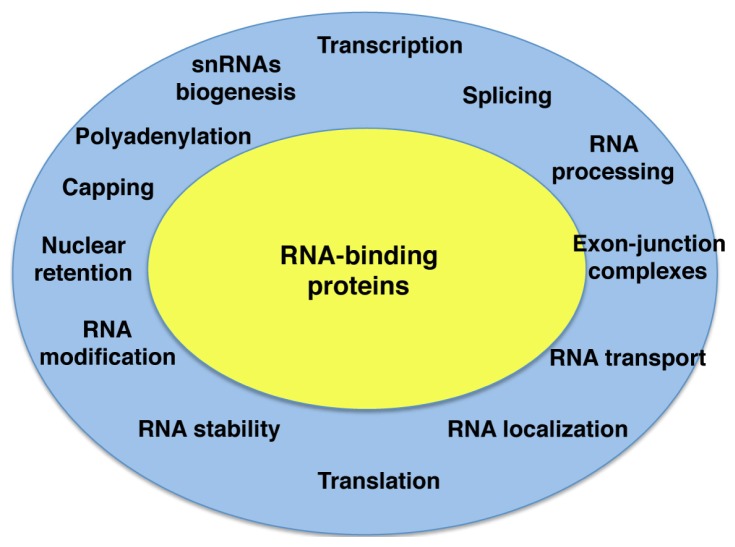
Gene expression control in eukaryotic cells. Each step of gene expression is controlled by RNA-binding proteins (**yellow**), such that altering one step affects the rest (**blue ring**) impacting on translation efficiency of mRNAs.

RNA plays a central role in gene expression. Within the cell RNA molecules are associated to RNA-binding proteins (RBPs) forming dynamic ribonucleoprotein particles (RNPs) that affect all steps of RNA metabolism [1]. RBPs assemble on nascent and processed mRNAs, governing gene regulation at post-transcriptional level in health and disease. Indeed, mutations affecting the function of RBPs cause several diseases [2]. RBPs are major components of the cell that control transcription, splicing, catalytic processing, transport, localization, translation or RNA stability processes (Figure 1). These steps in the RNA lifespan are closely connected to each other, such that altering one of them will affect the others.

**Figure 2:**
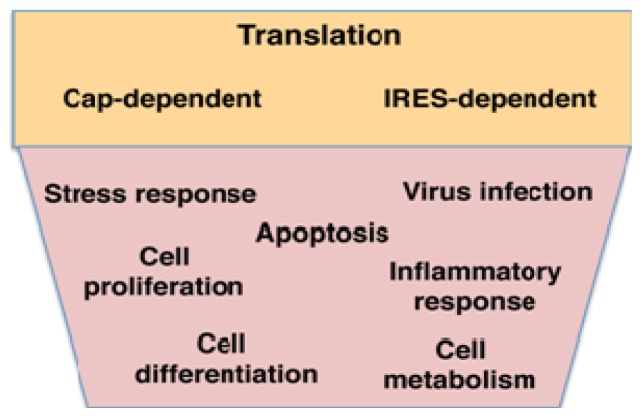
RNA-binding proteins control translation efficiency of mRNAs initiating protein synthesis by the conventional cap-dependent, or the alternative internal ribosome entry site (IRES)-dependent, mechanisms (**orange box**), impacting on cellular processes in cells undergoing normal growth as well as in response to environmental stresses (**pink box**).

RBPs often interact with the untranslated regions (UTRs) of mRNAs, which perform *cis*-acting regulatory functions in most cases and provide the landing site of many RBPs [3]. RBPs interacting with certain UTR structural elements or specific primary sequences play a pivotal role in the response of the cell to different environmental stresses, such as virus infections, heat or osmotic stress, nutrient deprivation and other stimuli that trigger apoptosis, inflammatory response, antiviral response, *etc.* (Figure 2) [4–6]. On the other hand, processes such as cell proliferation, cell death or cell differentiation occurring in healthy organisms also depend on RNA-protein interactions [7].

In response to distinct stresses, cells trigger a differential response that can displace the equilibrium towards cell survival or cell death. Key factors mediating this response are post-translational modification, relocalization, proteolysis or degradation of RBPs. A paradigmatic example of this response is observed in virus-infected cells [4]. Viral encoded proteases produced during picornavirus infection induce the proteolysis of a large number of host factors (Table 1) including splicing factors, RNA-processing proteins, RNA helicases or nuclear pore factors [8–21], leading to a redistribution of nuclear proteins to the cell cytoplasm. In addition, proteolytic cleavage of eukaryotic initiation factors (eIFs) [22–26] inhibits protein synthesis and in general, causes a shut-down of cellular gene expression. Specifically, cleavage of eIF4GI and PABP by picornavirus-encoded proteases induces the shut-off of cap-dependent translation in infected cells.

Traditional RBPs consist of modular RNA-binding domains (RBDs), typically RNA recognition motifs (RRM), heterogeneous nuclear ribonucleoprotein K-homology domains (KH), cold-shock domains (CSD), zinc finger domains (ZNF), double-stranded RNA-binding domains (dsRBD), Piwi/Argonaute/Zwile (PAZ) domain, RGG (Arg-Gly-Gly) box, DEAD/DEAH box, Sm domains and Pumilio/FBF (PUF or Pum-HD) domains [27]. Representative examples of RBPs are the polypyrimidin tract-binding protein (PTB), the hnRNP K, the upstream of *N*-ras (Unr), or the Zinc finger protein 9 (ZNF9). Among well characterized RBPs are hnRNPs, a large family of nuclear proteins (hnRNP A1 to hnRNP U) with RNA-binding domains and protein-protein binding motifs [28] that shuttle with the RNA from the nucleus to the cytoplasm and regulate transcription, splicing, RNA turnover and translation. PTB (also termed hnRNP I) is a protein with four RRMs that recognize U/C-rich sequences [29]. HnRNP K, PCBP1 (hnRNP E1) and PCBP2 (hnRNP E2) recognize polyr(C) regions and share the KH RNA-binding domain [30]. Unr is a cold-shock domain RBP that interacts with the poly(A)-binding protein (PABP) [31]. Zinc finger proteins, however, are DNA and RNA-binding proteins.

Given the many layers of post-transcriptional control operating in the cell, the number of factors that can be involved in various steps of RNA function is much larger than anticipated. Indeed, the recent development of methodologies aimed at searching for new RBPs has produced a catalogue of factors with RNA binding capacity [32], such as enzymes of intermediary metabolism among others. In the near future, in depth characterization will indicate whether these factors are passengers of RNP complexes or will provide evidences for the functional role of these factors in RNA-dependent pathways.

## 2. RNA-Binding Proteins and Translation Control

Most cellular mRNAs initiate translation by a mechanism that depends on the recognition of the m^7^G(5′)ppp(5′)N structure (termed cap) located at the 5′ end of most mRNAs [3]. In this mechanism, the 5′ cap structure of the mRNA is recognized by eIF4F, a complex composed of the cap-binding factor eIF4E, the scaffolding protein eIF4G and the RNA helicase eIF4A. The cap-binding capacity of eIF4E is regulated by phosphorylation level of eIF4E-binding proteins (eIF4E-BP 1-3). In turn, eIF4G interacts with eIF3 and PABP, the protein interacting with the poly(A) tail of the mRNA. In addition, the middle region of eIF4G around the first HEAT motif displays RNA binding capacity [33]. The thirteen multimeric factor eIF3 (eIF3a-eIF3m) is organized as a five-lobed structure [34]. Several subunits harbor RNA-binding domains (eIF3b and eIF3g) or have being involved in direct binding to mRNA (eIF3d). In addition, recent analysis of the interaction of eIF3 with a viral RNA has identified helix-loop-helix (HLH) motif [35] in eIF3a-c as the region responsible for RNA interaction.

On the other hand, the 40S ribosomal subunit with the ternary complex (TC) (consisting of the initiator methionyl-tRNA_i_ and eIF2-GTP) mediates the formation of the 43S complex that is recruited to the mRNA along with eIF1A, eIF1, eIF3, and possibly eIF5. Assembly of a competent 43S complex into mRNA bound to eIF4F is further stabilized by the interaction of eIF4G with PABP, and of eIF4B with eIF4A and PABP. The protein eIF4B harbors an RRM motif and a *C*-terminal Arginine-rich motif required for RNA-binding [36]. The 43S complex scans the 5′ UTR region of the mRNA until the first initiation codon in the proper context is encountered, leading to the formation of the 48S initiation complex. At this step, eIF1 ensures fidelity of initiation codon selection, discriminating against non-AUG and AUG codons located in poor context [37]. Furthermore, scanning of highly structured 5′ UTRs depends on the RNA helicase DHX29 [38]. Following eIF1 displacement, eIF5 mediates the hydrolysis of eIF2-bound GTP and eIF5B mediates joining of the 60S subunit yielding the 80S ribosome that gives rise to the start of polypeptide synthesis. For recent reviews on translation initiation see [3,6].

Cap-dependent translation initiation is inhibited under cellular stress conditions, such as viral infection or apoptosis [5,6,39]. Proteolysis of eIF4G and PABP and changes in the phosphorylation levels of eIF4E-binding proteins (eIF4E-BPs) severely compromise cap-dependent translation initiation in picornavirus infected cells [3]. These adverse situations, however, allow translation of some viral mRNAs and a subset of cellular mRNAs that evade translation shut-down, taking advantage of *cis*-acting regulatory elements known as internal ribosome entry site (IRES) elements. Translation of mRNAs bearing IRES elements, first reported in the genomic RNA of picornaviruses [40,41], is therefore resistant to cap-dependent shut-down.

Soon after picornavirus IRES disclosure, other viral RNAs [42,43] and cellular mRNAs possessing IRES elements were discovered due to their capacity to remain attached to polysomes under conditions that inhibit cap-dependent translation [44]. Cellular mRNAs bearing IRES elements do contain a cap at the 5′ end although they are translated at low levels, but can switch to an IRES-dependent mechanism when cap-dependent initiation is compromised [45]. In fact, there are examples where translation is increased (vimentin) and others where translation persists despite shut-down of cap-dependent translation (myc or nucleophosmin) [46]. The internal initiation process is assisted by RBPs, which are thought to facilitate the proper folding of the IRES region allowing the recruitment of the translation machinery [47]. The list of mRNAs that can be translated using cap-independent mechanisms is growing constantly [48–59]. For other IRES reports, see recent reviews [7,60]. Most IRES elements are located within the 5′ UTR of mRNAs. However, examples of IRES elements located within the coding sequence also exist [61–63]. In these cases, the polypeptide expressed from the internal initiation codon results in a shorter protein with different function [52,64]. Thereby, this manner of translation initiation opens new avenues for gene expression control.

Although the presence of IRES elements in viral RNAs is well established, data on some cellular elements has been a matter of debate due to the lack of appropriate controls performed to discard the presence of cryptic promoters or alternative splicing transcripts [65,66]. Indeed, conserved features of IRES elements in cellular mRNAs remain largely unknown since they differ in nucleotide sequence, RNA secondary structure and IRES *trans*-acting factors (ITAFs) requirement [67–69]. Moreover, the lack of conserved features hampers prediction of novel IRES elements using computational methods.

According to the minimal requirement of factors for internal initiation, viral IRES elements can be grouped into two categories. The first category, represented by the intergenic region (IGR) of dicistroviruses, includes those that do not need proteins to assemble the initiation complex. This unique class of IRES elements adopts a compact tertiary structure that functionally substitutes the initiator met-tRNA_i_ during internal initiation, driving protein synthesis without the help of eIFs at non-AUG codons [43,70,71]. The second category includes those elements that do need eIFs and RBPs to recruit the ribosome, such as picornavirus or cellular IRES elements [72–74]. In addition, distinct groups can be made within the second category, depending on the RNA structural organization and the proteins required for activity (see below).

## 3. RNA-Binding Proteins Modulating Viral IRES Activity

RNA structure plays a fundamental role in viral IRES-dependent translation initiation [75–77]. Structural and functional studies have shown that RNA structure of viral IRES elements is organized in phylogenetically conserved modules [42,78–80], suggesting a distribution of functions among the different domains of an IRES element [81,82]. Evidence for links between RNA structure and biological function is also supported by the conservation of structural motifs within IRES elements of highly variable genomes [83–86]. For the sake of brevity, we will discuss in this review the IRES elements located in the genome of picornavirus and hepacivirus.

### 3.1. RNA-Binding Proteins Modulating Picornavirus IRES Activity

Picornavirus IRES elements span from about 280 to 450 nucleotides upstream of the functional start codon [87]. Attending to RNA secondary structure organization and eIFs requirement, picornavirus IRES elements are grouped into four types, named I to IV [39]. Type I IRES [present in poliovirus (PV) and human rhinovirus (HRV)] and type II [present in encephalomyocarditis virus (EMCV) and foot-and-mouth disease virus (FMDV)] require the *C*-terminal region of eIF4G in addition to eIF4A, eIF2, and eIF3, but not eIF4E [88–91]. Furthermore, a differential requirement of eIF1 and eIF1A is needed to initiate at the second functional AUG of the FMDV IRES [92] which is, however, the most frequently used initiation codon on the viral RNA [93]. Translation initiation driven by type III IRES (present in Hepatitis A) does not require the full-length eIF4G [94]. On the other hand, type IV IRES (also termed HCV-like due to its similarity with hepatitis C virus (HCV) and pestivirus IRES elements [95,96] depend on eIF2 and eIF3, but they are eIF4G-independent. Interestingly, addition of specialized RBPs such as PTB or ITAF_45_ (also termed Ebp1) strongly stimulates complex formation in reconstitution assays [92,97,98].

The observation that ITAFs are RBPs previously identified as transcription regulators, splicing factors, RNA transport, RNA stability or translation control proteins (Table 2) suggests a complex network of interactions among gene expression pathways.

PTB was the first RBP identified as an ITAF using UV-crosslink assays conducted with radiolabelled IRES transcripts [99,100]. PTB is expressed in the cell as several isoforms. Interestingly, the expression pattern of the neural form of PTB was proposed to mediate cell-type IRES specificity of a neurotropic virus [115]. Many picornavirus IRES elements have two polypyrimidine tracts located at each end of the IRES region [39,116,117]. It appears that a single PTB molecule binds to the IRES, with RRM1-2 contacting the 3′ end and RRM3 the 5′ end of the IRES, constraining the RNA structure in a unique orientation [118]. However, subtle differences exist among IRES located in viral genomes belonging to the same virus family. Like other picornavirus IRES, Aichi virus (AV) IRES is enhanced by PTB, but this particular element depends on the RNA helicase DHX29 due to the sequestration of its initiation codon into a stable hairpin [97]. This example illustrates the different strategies that distinct viral IRES can use to capture the ribosomal subunits.

Nonetheless, the large amount of data obtained over the years on the mechanism of action of picornavirus IRES reveals that these regulatory elements are more complex than initially proposed. Proteomic studies based on mass spectrometry analysis of affinity purified RNPs assembled on tagged RNAs with cytoplasmic cell extracts have allowed the identification of RBPs interacting with several picornavirus IRES elements (Table 2). In support of the reliability of these approaches, proteins reported to interact with IRES elements by biochemical methods (for example, eIF4B and eIF3) were identified exclusively bound to the specific domains that contain their recognition motifs in both FMDV and the HCV IRES [102,119]. Various hnRNPs and RNA helicases have been reported to bind to picornavirus IRES elements. Proteins associated with viral IRES elements include the poly-r(C) binding protein (PCBP2), the SR splicing factor (SRp20) and the far upstream element binding protein 2 (FBP2). PCBP2 stimulates the IRES activity of PV, HRV and coxsackievirus B3 (CBV3) [104]; SRp20 up-regulates PV IRES-mediated translation via its interaction with PCBP2 following its relocalization to the cytoplasm in infected cells [105]; the nuclear protein FBP2 is a KH protein that shuttles to the cytoplasm in infected cells, negatively regulating enterovirus 71 (EV71) IRES activity [106].

Other examples concern proteins previously reported to perform a role distinct than translation control. A protein recently revealed as a factor regulating translation is Gemin5 [107], an abundant protein predominantly located in the cell cytoplasm. Gemin5 was reported to be the RNA-binding factor of the survival of motor neurons (SMN) complex that is responsible for the assembly of the seven member (Sm) proteins on snRNAs [120], the principal components of the splicing machinery. In addition, Gemin5 binds directly to the FMDV IRES element through its *C*-terminal region partially competing out PTB binding [121], a result that explains its negative effect on internal initiation of translation. On the other hand, recent studies have shown that PV IRES recruits glycyl-tRNA synthetase (GARS) taking advantage of the tRNA(Gly) anticodon stem-loop mimicry to the apical part of a conserved stem-loop adjacent to the binding site of eIF4G, enhancing IRES function at the step of the 48S initiation complex formation [108].

Given their modular organization, RBPs can recognize a large number of targets. This feature raises the possibility that binding of a particular RBP to certain RNAs could facilitate different sorts of regulation depending on the other partners of the complex. One example is Ebp1 (erbB-3-binding protein 1) identified in proteomic analysis bound to domain 3 of the FMDV IRES [102]. Ebp1 cooperates with PTB to stimulate FMDV IRES activity [92,98,117], but not EMCV IRES activity [103]. Protein-protein bridges could contribute to stimulate picornavirus IRES-dependent translation, as in the case of hnRNPs, helicases and Unr [109]. Conversely, heterodimers such us the NF45 (nuclear factor of activated T cells) with the double-stranded RNA-binding protein 76 (DRBP76, also termed NF90/NFAR-1) (DRBP76:NF45) repress HRV translation in neuronal cells [110]. In other cases, protein-protein association during mRNA transport, such as hnRNP U, hnRNP A/B, YB-1, or PTB [102,111,122] can explain the identification of cytoskeleton proteins by mass spectrometry of factors associated to viral IRES elements.

### 3.2. RNA-Binding Proteins Modulating Hepatitis C IRES Activity

The IRES element of HCV genome differs from picornavirus IRES elements in RNA structure and eIF4G requirement [39,123]. The HCV IRES is organized in three structural domains (designated II, III and IV) [124], although destabilization of domain IV is not detrimental to IRES function [42]. In the absence of eIFs, domain III can form a high-affinity complex with the 40S ribosomal subunit [125]. However, eIF3 and eIF2 are necessary for 48S initiation complex formation *in vitro* [72] despite the fact that HCV IRES activity has been shown to be partially resistant to eIF2 inactivation [126]. Recent cryoEM studies have contributed to the understanding of the interaction of eIF3 with the HCV IRES [35]. Mutations in the RNA-binding motif of eIF3a weaken eIF3 binding to the HCV IRES and the 40S ribosomal subunit, suppressing eIF2-dependent recognition of the start codon. Mutations in the eIF3c RNA-binding motif also reduce 40S ribosomal subunit binding to eIF3 and inhibit eIF5B-dependent steps downstream of start codon recognition.

In addition to eIF3 and the ternary complex, a few RBPs acting as ITAFs are shared between HCV and picornavirus IRES elements (PTB, PCBP2, Nucleolin, Gemin5, Unr, hnRNPA1/A2, La autoantigen (La) and NS1-associated protein (NSAP1, also known as hnRNP D) [101,107,112–114]). Whether these RBPs modulate translation initiation promoted by other viral IRES remains to be elucidated.

Both picornavirus and HCV IRES-dependent translation are synergistically enhanced by the 3′ UTR of the viral genome [127–129], consistent with a functional link between the 5′ and 3′ ends of the viral RNA. In picornavirus RNAs, the 3′ UTR is composed of two stem-loops and a short poly(A) tail that are required for virus multiplication. In contrast, the HCV viral RNA possesses a poly(U) tract and a complex RNA structure located near the 3′ end. Bridging 5′ and 3′ ends of viral RNAs involves direct RNA-RNA contacts and RNA-protein interactions [130,131]. Accordingly, riboproteomic procedures on RNAs with two distant *cis*-acting regions identified factors mediating the formation of functional bridges between mRNA regions. This was the case of RNPs assembled on tagged RNAs that contained both the IRES and the 3′ UTR of HCV [111]. One of the identified proteins, the insulin-like growth factor II mRNA-binding protein 1 (IGF2BP1), coimmunoprecipitates with eIF3 and the 40S ribosomal subunit, suggesting that it enhances HCV IRES-dependent translation by recruiting the ribosomal subunits to a pseudo-circularized RNA. Recent studies have proposed that 3′ UTR interaction with the ribosomal subunit retains ribosome complexes during translation termination, facilitating efficient initiation of subsequent rounds of translation [132].

## 4. RNA-Binding Proteins Controlling Cellular IRES Activity

Cellular IRES elements are typically present in mRNAs encoding stress response proteins, such as those needed during nutrient deprivation, temperature shock, hibernation, hypoxia, cell cycle arrest, or apoptosis [5,7]. Hence, they have evolved mechanisms to evade global repression of translation. The study of some cellular IRES elements has shown that ITAFs facilitate the binding of the mRNA to ribosomal 40S subunits, in conjunction with eIF2, eIF3, eIF4F, and PABP [133–136]. Accordingly, changes in ITAFs abundance, post-translational modifications or subcellular localization, modulate internal initiation of translation during different stress conditions [137].

Multifunctional RBPs interact with various IRES elements, suggesting a mechanism for the coordinated regulation of translation initiation of a subset of mRNAs bound by shuttling proteins, such as hnRNPs and splicing factors (Table 3). Nuclear proteins found in complexes associated with various gene expression steps involving RNA molecules (PTB, the splicing-factor related protein proline and glutamine-rich SFPQ/PSF, the non-POU domain-containing octamer binding nuclear RNA binding protein (nonO/p54nrb), PCBP2, or HuR) control the expression of lymphoid enhancer binding factor (LEF1), c-myc, CDK11, or p53 [136,138–141]. However, there are other cases where specific RBPs (such as mouse hnRNP Q and FMRP) are reported to control a reduced number of IRES elements [142,143]. This differential regulation suggests that specialized RBPs might exert their function in translation control by binding to the IRES region of specific cellular mRNAs during splicing complex assembly before nuclear export. It remains to be determined whether this is the result of a specialized activity or if it reflects the lack of sufficient detailed studies. Below, we discuss the capacity of various RBPs to control cellular IRES elements driving the expression of proteins grouped according to their function in the cell. In this process, deleting the content is not recommended.

### 4.1. ITAFs Controlling the Expression of Cell Proliferation Proteins

PTB stimulates translation driven by IRES elements located in mRNAs encoding proteins of the myc family controlling cell growth, the tumor suppressor gene *p53* as well as other factors involved in apoptosis and nutrient deprivation [135,136,144]. A complex formed by Annexin A2, PSF and PTB binds and stimulates p53 IRES in the presence of calcium ions [139]. The unr, c-myc, CDK11, and serine/threonine-protein kinase PITSLREp58 IRES elements are activated during mitosis [140,146], a cell cycle phase where cap-dependent translation is compromised. Protein-protein interaction and/or coordinated RNA-proteins complex assembly influence internal initiation, as shown in the case of IRES activity of c-myc and PITSLRE mRNAs, whose function depends on the Unr-partners, hnRNP K, PCBP1-2, or hnRNP C1-2, respectively [141,147]. On the other hand, stress-dependent modifications or relocalization of hnRNP A1 mediates internal initiation of c-myc, unr, cyclin D1, or sterol-regulatory-element-binding protein 1 (SREBP-1a) mRNAs [148,149].

Translation of specific mRNAs in cells with quiescent v-akt murine thymoma viral oncogene homolog 1 (AKT) kinase maintains the levels of proteins involved in cell cycle progression when eIF4E-mediated (cap-dependent) translation is inhibited. This pathway is dependent on SAPK2/p38-mediated activation of IRES-dependent initiation of the cyclin D1 and c-myc mRNAs [152]. Inhibition of SAPK2/p38 in glioblastoma multiforme cells reduces rapamycin-induced IRES-mediated translation initiation of cyclin D1 and c-myc, resulting in G1 arrest and inhibition of tumor growth.

### 4.2. ITAFs Controlling Translation of Pro-apoptotic and Pro-survival mRNAs

IRES located in mRNAs encoding proteins synthesized under apoptosis such as the apoptotic protease activating factor 1 (Apaf-1), and BCL2-associated athanogene (BAG-1), are also responsive to PTB [145]. In particular, IRES activity of Apaf-1 mRNA is regulated via PTB and Unr [74]. However, during apoptosis the Apaf-1 IRES is activated while the X-linked inhibitor of apoptosis protein (XIAP) is inhibited [161]. It has been reported that relocalization of hnRNP A1 mediates internal initiation of Apaf-1 and XIAP [150,151]. Other proteins such as DAP5 and HuR exert a stimulatory role on apoptotic mRNAs [153,154].

With the exception of pyrimidine tracts, no distinctive RNA motifs that can be used to predict the binding of RBPs are apparent in cellular IRES elements. Yet, cellular IRES with high AU content, such as XIAP, depend on NF45 [157], since cells deficient in NF45 exhibit reduced IRES-mediated translation of XIAP and cellular inhibitor of apoptosis protein 1 (cIAP1) mRNAs that, in turn, leads to dysregulated expression of survivin and cyclin E.

Although most ITAFs stimulate translation, a few cases where RBPs repress translation have also been reported. IRES-dependent translation of anti-apoptotic proteins XIAP and Bcl-X is repressed by the tumor suppressor programmed cell death 4 (PDCD4), a factor that sequesters eIF4A and, thus, inhibits formation of the 48S translation initiation complex [162]. Phosphorylation of PDCD4 by activated S6K2 leads to the degradation of PDCD4, stimulating XIAP and Bcl-x(L) translation.

### 4.3. ITAFs Controlling Response to Nutrient Starvation, ER Stress, or Growth Factors

As in the case of IRES elements controlling the expression of cell proliferation proteins, stress-dependent relocalization of hnRNP A1 also mediates internal initiation of vascular endothelial growth factor (VEGF) and fibroblast growth factor (FGF-2) [151]. However, a negative effect of DKC1 on the VEGF IRES has been recently reported [163], likely due to a reduction on ribosome availability. HuR was found to negatively affect translation of the IGF1 receptor or the thrombomodulin (TM) endothelial cell receptor [159] by yet unknown mechanisms. A different case of a repressor ITAF is the DEAD-box RNA helicase 6 (DDX6) [160]. DDX6 inhibits VEGF IRES-mediated translation in normoxic MCF-7 extract. However, under hypoxia the level of DDX6 declines and its interaction with VEGF mRNA is diminished *in vivo*. In addition, DDX6 knockdown cells show increased secretion of VEGF.

The death-associated protein (DAP5), NF45, G-rich RNA sequence binding factor (GRSF-1), fragile-X mental retardation protein (FMRP), heterogeneous nuclear ribonucleoprotein D-like protein (JKTBP1) and zinc-finger protein ZNF9 are specific for some IRES elements such as DAP5/p97, cIAP1, ODC [155,156,158,164]. Moreover, circadian expression of mouse PERIOD1 (mPER1) is regulated by rhythmic translational control of mPer1 mRNA, together with transcriptional modulation, via an IRES element along with the mouse *trans*-acting factor mhnRNP Q [143]. The rate of IRES-mediated translation exhibits phase-dependent characteristics through rhythmic interactions between mPer1 mRNA and mhnRNP Q.

In summary, the data available so far indicate that individual mRNAs exploit the activity of multifunctional RBPs to overcome the global repression of protein synthesis.

## 5. Concluding Remarks and Perspectives

ITAFs are RBPs that activate or repress the expression of proteins critical in the cellular response to growth, nutritional, environmental and proliferation signals. It has been proposed that the main role of RBPs is to remodel the mRNA structure in a way that enhances its affinity for components of the translation machinery, or that they substitute some canonical eIFs providing functional bridges between the mRNA and the ribosomal subunits. Elucidating the function of ITAFs demands a deep understanding of their RNA targets and their protein partners with potential modifications. Yet, a subset of IRES elements exhibiting different structural organization interacts with the ribosomal protein RpS25 [165], consistent with the observation that two of these IRES elements (HCV and the dicistrovirus IGR) induce similar conformational changes in the 40S ribosomal subunit despite their different RNA structural organization [166]. Indeed, the IRES property of interacting with the ribosomal RNA [167,168] is an attractive idea that is also consistent with the finding that IRES activity is sensitive to changes in the ribosome composition [169–171]. In the near future, the characterization at the molecular level of more IRES elements will shed new light on the understanding of the strategies used to recruit the translation machinery. In turn, this will open new avenues to predict novel regulatory elements using this specialized mechanism of translation initiation.

## Figures and Tables

**Table 1:** RNA-binding proteins proteolyzed in picornavirus infected cells.

Protein	Role on gene expression	Reference
eIF4GI, eIF4GII	Cap-dependent translation	[4,26]
eIF4A	Cap-dependent translation	[22]
PABP	Cap-dependent translation	[24,25]
eIF3a	Cap-dependent translation	[24]
eIF5B	Cap-dependent translation	[23]
PTB	IRES-dependent translation	[24]
PCBP2	Switch from translation to RNA replication	[10]
Sam68	Signal transduction and activation of RNA	[20]
Gemin3	RNA helicase, U snRNP assembly	[8]
Gemin5	SMN complex, IRES-dependent repressor	[18]
RIG-1	Antiviral response	[11]
MAVS, TRIF	Antiviral response	[9]
G3BP	Stress granules assembly	[19]
Nup62, Nup98, Nup153	Nuclear pore	[14,17,21]
CstF-64	Cellular polyadenylation	[12]
FBP2/KSRP	Transcription activation, mRNA decay	[13]
La	RNA polymerase III transcription	[15]
AUF1	mRNA stability	[16]

**Table 2:** RNA-binding proteins modulating viral IRES activity.

Protein function	Activity on IRES *	Reference
DHX29: RNA helicase, translation	Stimulation	[97]
PTB: Splicing, RNA stability, RNA localization	Stimulation	[99–101]
Ebp1: Transcription regulator	Stimulation	[102,103]
PCBP2: RNA stability, translation	Stimulation	[104]
SRp20: Splicing	Stimulation	[105]
FBP2: RNA stability	Repressor	[106]
Gemin5: snRNAs biogenesis, translation	Repressor	[107]
GARS: Glycyl-tRNA synthetase	Stimulation	[108]
Unr: Translation control	Stimulation	[109]
DRBP76:NF45: Transcription, RNA stability	Repressor	[110]
IGF2BP1/IMP1: RNA stability, translation	Stimulation	[111]
La: Transcription, translation control	Stimulation	[112]
NSAP1/hnRNP Q: RNA processing, translation	Stimulation	[113]
hnRNP L,D: RNA stability, translation	Stimulation	[114]

* Proteins interacting with viral IRES but with no known activity on translation initiation have not been included.

**Table 3:** RNA-binding proteins m odulating cellular IRES.

ITAF	Reference
PTB	[74,133,134,136,140,144,145]
YB-BP1, GRSF	[133]
Unr	[74,146]
SFPQ/PSF, nonO/p54nrb	[138]
PSF/annexin 2	[139]
PCBP1/2	[147]
hnRNP C1/C2	[141,147]
hnRNP A1/A2	[148–151]
SAPK2/p38	[152]
DAP5	[62,153–155]
ZNF9	[156]
NF45	[157,158]
HuR	[154,159]
hnRNP L	[134]
hnRNP Q	[143]
FMRP	[142]
DDX6	[160]
